# Carbon-Based Adsorbents for Microplastic Removal from Wastewater

**DOI:** 10.3390/ma17225428

**Published:** 2024-11-06

**Authors:** Nii Ashitey Anuwa-Amarh, Melike Dizbay-Onat, Kaushik Venkiteshwaran, Shenghua Wu

**Affiliations:** 1 William B. Burnsed, Jr. Department of Mechanical, Aerospace, and Biomedical Engineering, University of South Alabama, Mobile, AL 36688, USA; na2423@jagmail.southalabama.edu; 2 Department of Civil, Coastal, and Environmental Engineering, University of South Alabama, Mobile, AL 36688, USA; kvenkiteshwaran@southalabama.edu (K.V.); shenghuawu@southalabama.edu (S.W.)

**Keywords:** microplastic removal, wastewater, carbon-based adsorbents

## Abstract

Plastics are widely used across various industries due to their flexibility, cost-effectiveness, and durability. This extensive use has resulted in significant plastic pollution, with microplastics (MPs) becoming pervasive contaminants in water bodies worldwide, adversely affecting aquatic ecosystems and human health. This review explores the surface characteristics of carbon-based adsorbents, including biochar, activated carbon, carbon nanotubes (CNTs), and graphene, and their influence on MP removal efficiency. Key surface characteristics such as the carbon content, surface area, pore size, and particle size of adsorbents influenced adsorption efficiency. Additionally, hydrophobic interaction, van der Waals forces, π–π interactions and electrostatic interaction were found to be mechanisms by which microplastics are trapped onto adsorbents. Modified biochar and activated carbon demonstrated high adsorption efficiencies, while CNTs and graphene, with their high carbon contents and well-defined mesopores, showed outstanding performance in MP removal. Although a high surface area was generally associated with better adsorption performance, modifications significantly enhanced efficiency regardless of the initial surface area. This review emphasizes the importance of understanding the relationship between surface characteristics and adsorption efficiency to develop optimized adsorbents for MP removal from wastewater. However, challenges such as the lack of standardized testing methods, variability in biochar performance, and the high cost of regenerating carbon adsorbents remain. Future research should focus on developing cost-effective production methods, optimizing biochar production, and exploring advanced modifications to broaden the application of carbon adsorbents. Integrating advanced adsorbents into existing water treatment systems could further enhance MP removal efficiency. Addressing these challenges can improve the effectiveness and scalability of carbon-based adsorbents, significantly contributing to the mitigation of microplastic pollution in wastewater.

## 1. Introduction

Plastics are extensively used across various daily activities such as packaging, building, textiles, medicine, farming, electronics production, and more, due to their impressive flexibility, cost-effectiveness, adaptability, outstanding resistance, and lightweight nature [1,2]. In 2020, global plastic production surged to an immense 367 million tons, with a projected 12,000 million tons being produced by 2050 [3,4]. Despite the significant amount of plastic being generated, global plastic recycling rates have remained at around 9% since 1950, leading to the majority of it entering rivers and oceans [1,5,6]. Microplastics (MPs) are described as plastic materials which are smaller than 5 mm in size [7,8]. MPs are formed from larger plastic products, such as textiles and cosmetics, which degrade into smaller micro-particles (100 nm to 5 mm), influenced by factors like temperature, physical/chemical interactions and UV radiation [4,6,9]. An estimated 51 trillion plastic particles are dispersed throughout surface waters worldwide [10,11,12,13]. These particles can be found in water bodies all over the world and are a significant pollutant for humans and the environment at large [14]. Their small size allows them to evade many filtration systems, accumulate in water bodies and pose severe threats to aquatic life, terrestrial animals and human health through the food chain [15,16]. A literature survey of over 40 operational municipals and industrial wastewater treatment plants across the world, with various combinations of primary treatment, secondary treatment and tertiary treatment, revealed that a significant amount of MPs are removed through existing treatment processes [17,18,19,20].

**Figure 1:**
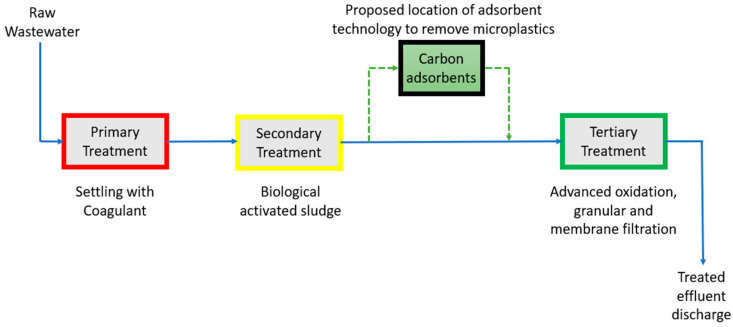
Schematic showing the different stages at a typical wastewater treatment plant.

The primary treatment process based on physical mechanisms is considered the first barrier to removing MPs in wastewater treatment plants [21]. The use of a primary settling tank with coagulant addition was the most frequently implemented primary treatment method [22]. MP concentration decreased by 4 to 99% compared to the concentration in the influent, and was removed as primary settled sludge [20]. Biologically activated sludge treatment is the most common secondary treatment step in most wastewater treatment plants [23]. The removal efficiency after secondary treatment processes ranged from 20 to 96% compared to the primary influent [18,19,20]. More than half of the surveyed wastewater treatment plants employed tertiary treatment processes such as advanced oxidation and granular or membrane filtration [24]. After tertiary treatment processes, MP abundance further decreased in most of the investigated wastewater treatment plants, with efficiency ranging between 50 and 99.6% compared to the abundance in the influent [19,20]. The wide range of removal efficiencies observed across different treatment processes can be due to several factors, including the dose of chemical coagulant, the type of treatment used, the characteristics of the MPs, and operational and environmental conditions [17,18,19]. Despite removal, a significant proportion (1 to 50%) of total MPs can still be discharged through the plant effluent [25]. A majority (80 to 90%) of these MPs are neutrally buoyant fibers of <1 mm particle size [17]. These smaller MPs are often secondary microplastics generated from the breakdown of primary (larger) microplastics as they pass through the primary and secondary treatment processes, as shown in Figure 1 [20].

**Figure 2:**
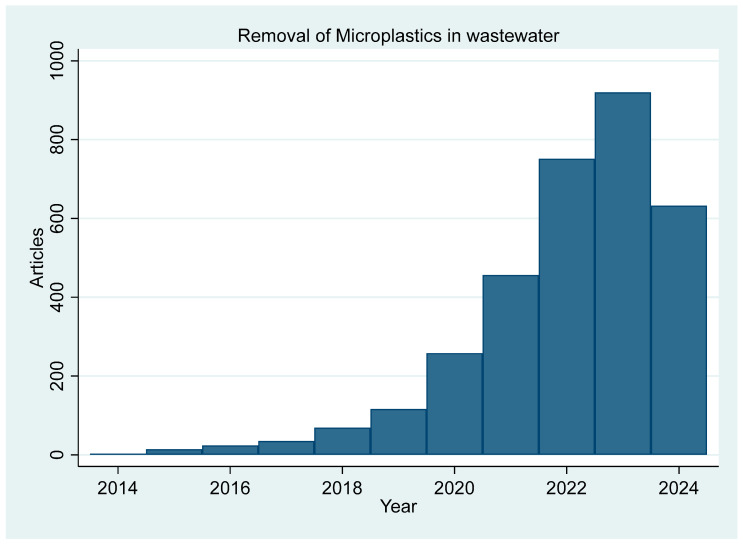
Related research articles on the removal of MPs in wastewater published in *ScienceDirect* between 2014 and 2024.

To further improve MP removal and prevent environmental release, several strategies have been investigated in the last decade. From 2014 to date, 3278 research articles have been published which targeted the removal of MPs in wastewater, as seen in Figure 2. This research area started slowly in late 2014 and early 2015 and gained popularity in the years since 2020.

Strategies for addressing microplastic (MP) removal from wastewater treatment plants (WWTPs) can be broadly classified into three categories: physical, chemical, and biological methods. Physical methods encompass a variety of techniques such as adsorption, membrane separation, filtration, screening, electrocoagulation, and flotation [26,27,28,29]. Mechanical systems with fine mesh filters have been employed to capture larger MPs from water sources. However, the efficiency in capturing the smallest particles varies, and often, additional methods are required to enhance removal [30]. Carbon-based adsorbents have high mechanical and chemical stability as well as a promising ease of regeneration and reusability [31]. Membrane filtering uses technologies like nanofiltration and reverse osmosis, especially in areas requiring high-purity water, such as drinking water treatment plants or industrial processes [32,33,34]. These systems are efficient at removing a wide range of contaminants, including MPs.

Chemical approaches like photocatalysis and peroxide oxidation are pivotal in MP removal processes [35]. Chemical methods for MP removal are relatively rare and are mainly utilized in research settings or specialized applications. While there has been exploration into using oxidizing agents to break down MPs, their widespread adoption is limited by concerns about potential by-products and sustainability [36]. Similarly, biological methods, which are primarily in the research or pilot stages, have not yet been widely implemented [37].

Among the various available techniques for capturing MPs from wastewater, adsorption stands out as an economical, straightforward, reliable, and effective method. It offers several advantages, including simplicity, efficiency, and reusability, making it an attractive option for precise MP elimination [38]. Another notable strength is the flexibility to choose adsorbents tailored to specific pollutants, enhancing the technique’s efficacy. A diverse array of materials has been utilized for MP remediation, ranging from biochar and activated carbon to sponges, aerogels, and various nanoparticles [39].

Carbon-based adsorbents, including granular activated carbon, biochar, and carbon nanotubes, are notably praised for their cost-effectiveness and high adsorption efficiency for water pollutants. Surface characteristics of carbon-based adsorbents, such as surface area, porosity, shape, and surface charge, play a crucial role in their removal efficiency. For instance, surface area is an important factor in adsorption because it provides many sites for attachment. High surface area is directly proportional to high adsorption pollutant removal efficiency [40]. Smaller sizes of particles have a larger surface area-to-volume ratio, which enhances adsorption capacity. Pore size distribution is also an important parameter for pollutant adsorption. Despite their potential, there is a notable gap in the research concerning detailed information on important surface characteristics of carbon-based adsorbents and their MP removal efficiency [41].

**Figure 3:**
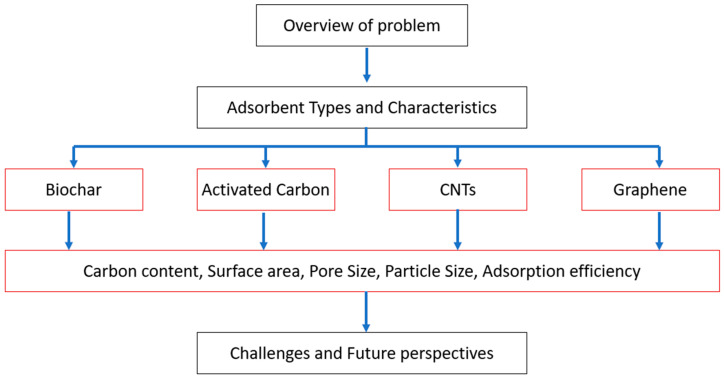
Structure of the review paper.

The goal of this review is to examine the relationships between adsorption efficiency and surface characteristics such as surface area, pore size, particle size, and carbon content. This integrated approach fills a critical research gap. This review offers a holistic understanding of how specific surface characteristics impact adsorption efficiency, providing new perspectives that can guide targeted research and practical applications. In contrast, other reviews often do not detail the role of individual surface characteristics, leading to fragmented information that makes it difficult to grasp the full picture. By integrating existing knowledge and highlighting the relationship between adsorbent properties and microplastic removal, our review offers valuable insights for researchers and practitioners in the field of wastewater treatment. Figure 3 shows the basic structure of the manuscript.

An overview of the problem highlights the problem of microplastic pollution in water bodies and demonstrates why we urgently need effective methods to remove these pollutants. This is followed by an investigation into the different types of carbon-based adsorbents that can help, like biochar, activated carbon, CNTs, and graphene. Each adsorbent type is analyzed for its detailed characteristics, including carbon content, surface area, pore size, particle size, and adsorption efficiency. This analysis helps in understanding the properties and effectiveness of each adsorbent for MP removal. The final section addresses challenges and future perspectives, discussing the current challenges in utilizing these adsorbents as well as the future research directions and potential advancements.

## 2. Synthesis and General Characterization of Carbon-Based Adsorbents

**Figure 4:**
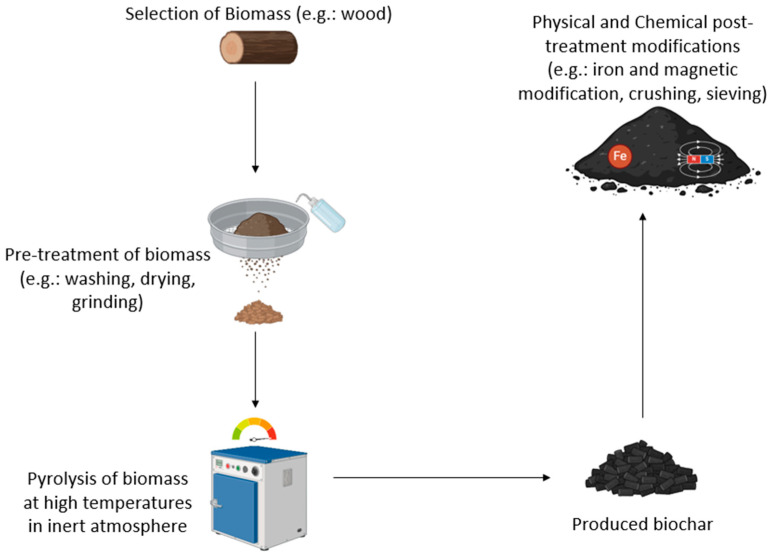
The synthesis process of carbon-based adsorbents.

The synthesis of carbon-based adsorbents typically involves the pyrolysis of various biomass feedstocks under controlled conditions, as seen in Figure 4. The key steps in the synthesis process are as follows:

- Selection of biomass feedstock: Common feedstocks include agricultural residues, wood, and aquatic plants. For example, corn straw and hardwood biochar were selected to be used as adsorbents [42].
- Pre-treatment: The biomass is often washed, dried, and ground to a uniform particle size to ensure consistent pyrolysis. For instance, magnetic biochar was soaked in a solution to pretreat the sawdust [43].
- Pyrolysis: The prepared biomass is subjected to pyrolysis, which involves heating in an inert atmosphere at temperatures ranging from 300 °C to 900 °C. The pyrolysis temperature and duration are critical factors that determine the surface area, porosity, and particle size of the biochar. For example, rape straw biochar was pyrolyzed under a nitrogen gas atmosphere at 800 °C for two hours [44].
- Post-treatment modifications: To enhance the adsorption capacity, carbon adsorbents such as biochar and activated carbon can undergo various physical and chemical modifications such as crushing, sieving, polymer coating, and iron and magnetic modification [44,45].

The performance of carbon-based adsorbents is highly dependent on their physical and chemical properties, which are characterized using several techniques.

- Surface area and porosity: Brunauer–Emmett–Teller (BET) surface area analysis and the mercury porosimeter method provide information on the surface area and pore size [43,46,47,48].
- Morphology: Scanning Electron Microscopy (SEM) and Transmission Electron Microscopy (TEM) are used to examine the surface morphology and pore structure of the biochar. These techniques provide insights into the physical structure and the presence of functional groups on the surface [43,44,47,48,49,50].
- Elemental composition: Energy Dispersive X-ray Spectroscopy (EDS) and elemental analyzers help to determine the elemental composition of the biochar, including the presence of elements like carbon, oxygen, and any incorporated metals [43,44,50].
- Functional groups: Fourier Transform Infrared Spectroscopy (FTIR) identifies the functional groups present on the biochar surface, which are critical for adsorption processes [43,46,48,49,50].
- Magnetic properties: For magnetic biochar, a Vibrating Sample Magnetometer (VSM) measures the magnetic properties, which are important for the separation of the adsorbent from aqueous solutions [43,44,49].

## 3. Effects of Surface Characteristics of Carbon-Based Adsorbents on MP Removal

### 3.1. Biochar

**Figure 5:**
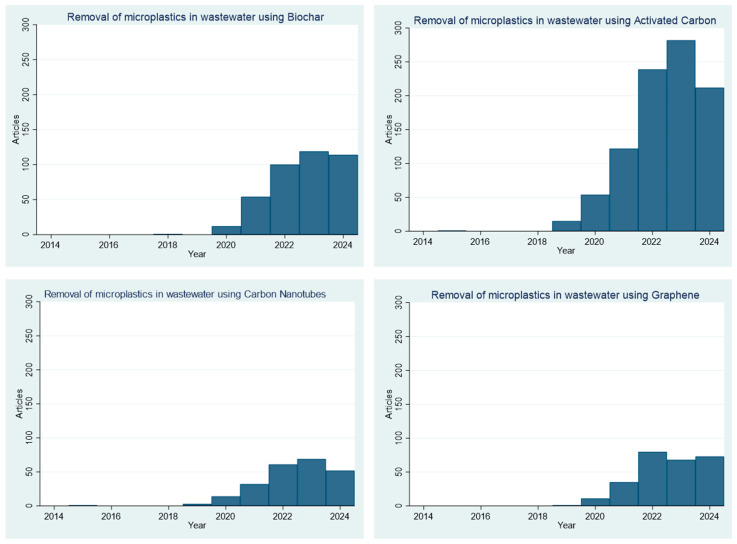
Related research articles on the removal of MPs in wastewater using carbon adsorbents published in *ScienceDirect* between 2014 and 2024.

Biochar is obtained by biomass pyrolysis, whereby solid matter from living organisms is heated in an oxygen-limited environment. This process results in the formation of solid carbon-rich products known as char [51,52,53]. Within the last 10 years, 12% of published papers targeted the method of using biochar as an adsorbent to remove MPs from wastewater, and this research actively started around 2020, as seen in Figure 5. Biochar primarily adsorbs MPs through physical adsorption and pore-filling mechanisms, whereby the porous structure and high surface area of biochar provide various sites to trap MPs. The biochar surface also forms hydrogen bonds and π–π interactions which interact with MPs, enhancing adsorption [43].

#### 3.1.1. Carbon Content of Biochar

Out of 400 research papers reviewed, only eight papers specifically showed the elemental carbon percentage present in the adsorbent. It was observed that a high carbon content of the biochar resulted in high adsorption efficiency, as seen in Table 1. For corn straw biochar, despite slight variations in carbon content, the efficiency remained consistently high at 95%.

Walnut shell biochar with a high carbon content of 86.44% showed lower efficiency in its unmodified form but significantly higher efficiency when modified [54]. It was also observed that biochar with moderate carbon content revealed a significantly higher efficiency. For example, sawdust biochar with a moderate carbon content between 65.03% and 69.29% showed very high adsorption efficiencies between 94.81% and 99.46% when modified [43]. Rape straw biochar with 66.99% carbon content showed a high efficiency of 91.2% to 95% [44]. Biochar with a lower carbon content also showed high removal efficiency with modifications. For example, aquatic-plant-derived biochar showed increasing efficiency with increasing carbon content, from 68.2 mg/g at 45% carbon to 89.06 mg/g at 50% carbon [55]. Also, polymer-coated magnetic activated biochar with a lower carbon content of 42.8% showed a very high efficiency of 99 mg/g to 100 mg/g [45]. Corncob biochar showed an increasing efficiency with increasing carbon content in its non-oxidized form. However, for oxidized biochar, there was a slight decrease in carbon content with an increase in efficiency [46].

The carbon content varied significantly with the type of raw material used. For example, corn straw biochar had a carbon content ranging from 68.7% to 81.3% depending on the carbonization temperature. Corncob biochar exhibited a higher carbon content, reaching up to 81.2% at 900 °C, though oxidized corncob biochar showed a reduced carbon content of 65.3%. Walnut shell biochar, particularly when iron-modified, saw a reduction in carbon content from 86.44% to 59.27%. Secondly, the efficiency of biochar in adsorbing pollutants generally improved with increasing temperature, which also influenced the carbon content. For example, corn straw biochar maintained a consistent adsorption efficiency of 95% from 300 °C to 500 °C, while the carbon content decreased from 81.3% at 400 °C to 68.7% at 500 °C. Also, corncob biochar showed an increased adsorption capacity (from 12 mg/g to 18 mg/g) as the temperature rose from 500 °C to 900 °C, although this was accompanied by a decrease in carbon content, particularly in oxidized samples. Lastly, modifications to the raw biochar material significantly altered its properties. For example, modified biochar such as iron-modified walnut shell exhibited an enhanced adsorption efficiency, but at the cost of reduced carbon content. This highlights why varying carbon content adsorption efficiencies were reported, as they were influenced by the raw material, carbonization temperature, and modifications.

#### 3.1.2. Surface Area of Biochar

Nine papers revealed the surface areas of the adsorbent. For biochar, a higher surface area did not always correlate with higher adsorption efficiency, as seen in Table 1. For example, corn straw biochar @ 300 °C with a high surface area of 808.3 m^2^/g had a lower efficiency of 70.0% compared to corn straw biochar @ 400 °C with a surface area of 609.0 m^2^/g and a higher efficiency of 81.3% [42]. Similarly, sawdust biochar @ 550 °C with no modifications had the highest surface area of 405.76 m^2^/g but the lowest efficiency of 25.89% among its variants [43]. It was also observed that modifications could enhance the efficiency of biochar irrespective of the surface area. Mg and Zn modifications to sawdust biochar resulted in higher efficiencies of 98.75% to 99.46% with surface areas of 265.74 m^2^/g and 329.87 m^2^/g, respectively, compared to the unmodified version with a surface area of 405.76 m^2^/g and a much lower efficiency of 25.89% [43].

Corncob biochar showed a clear trend, in which the surface area increased with pyrolysis temperature and oxidation, resulting in higher adsorption capacities. For example, oxidized corncob biochar @ 900 °C had the highest surface area of 60.8 m^2^/g and the highest adsorption capacity of 18 mg/g [46]. It was also observed that biochar with a lower surface area could still have a high adsorption efficiency due to other factors. For example, banana peel biochar with a very low surface area of 12.277 m^2^/g still had a high efficiency of 92.16% under conditions where the microplastic size ranged from 150 µm to 300 µm, a 6 cm bed height, there was a 3 mL/min flow rate, and the inlet concentration was 0.05 g/L [58]. It can be seen that the relationship between surface area and biochar efficiency is complex and influenced by multiple factors. While surface area is an important factor, it is not the only determinant of biochar efficiency. Other factors such as biochar composition, modifications, pore structure, and pyrolysis temperature also play significant roles.

Modifications (e.g., magnetic, Mg, Zn) can significantly enhance adsorption efficiency, sometimes even compensating for lower surface areas. For oxidized biochar, there is a more consistent positive correlation between surface area and adsorption capacity.

#### 3.1.3. Pore Size of the Biochar

Five papers mentioned the pore sizes of the adsorbent. The biochar was generally made up of both micropores and mesopores, as seen in Table 1. Biochar with mesopores (2–50 nm) showed higher efficiency.

For example, magnetic sawdust biochar with mesopores (3.81 nm and 3.83 nm) had high efficiencies of 94.81% to 99.46% [43]. Walnut shell biochar with mesopores (3.17 nm to 3.23 nm) also showed improved efficiency upon iron modification [54]. Magnetic activated biochar with mesopores of 9.82 nm showed high efficiency (96 mg/g to 100 mg/g) [45]. Biochar with micropores (<2 nm) showed lower efficiency, as seen in the sawdust biochar with micropores (1.86 nm), which had significantly lower efficiency (25.89%) compared to the mesoporous alternatives [43]. Corncob biochar with micropores (7.07 nm to 9.79 nm) showed moderate adsorption capacities of 12 mg/g to 18 mg/g [46]. Biochar with a mixture of micropores and mesopores revealed a variable adsorption efficiency. For example, aquatic plant-derived biochar with mixed pores between 13.04 nm and 30.13 nm had high efficiencies from 68.2 mg/g to 89.06 mg/g, increasing with temperature [55]. Biochar with mesopores tended to have higher adsorption efficiencies for microplastic removal compared to those with micropores. Mixed pore structures can also be effective, indicating that an optimal pore size distribution may enhance adsorption performance. Modifications that introduce or enhance mesopores contribute significantly to improved efficiency.

#### 3.1.4. Particle Sizes of Biochar

Seven papers mentioned the particle sizes of the carbon adsorbent. It can be observed that smaller particle sizes for biochar generally result in higher adsorption efficiencies, as seen in Table 1. For example, banana peel biochar with a particle size between 0.15 mm and 0.5 mm had a high adsorption efficiency of 92.16% [58]. Corncob biochar with particle sizes of around 0.15 mm and 100 nm showed an increasing adsorption capability with smaller particle sizes and higher temperatures [46]. Larger particle sizes showed a varying adsorption efficiency. Corn straw biochar (0.6–0.7 mm) showed a varying efficiency between 68.7% and 81.3%, influenced by pyrolysis temperature [42]. Aquatic-plant-derived biochar (1–2 mm) revealed good efficiency (68.2 mg/g to 89.06 mg/g), which increased with temperature [55]. Modifications (e.g., oxidation, magnetic, polymer coating) significantly enhanced the adsorption efficiency of biochar, even for larger particles. For example, polymer-coated magnetic activated biochar (20.68 nm) had an efficiency of 99 mg/g to 100 mg/g [45]. In as much as these observations were made, some papers did not take into account the particle size of the adsorbents.

Biochar has varying particle sizes due to the type of biomass used, the conditions under which it is produced, and any modifications applied. Different biomass sources like wood, agricultural residue, or aquatic plant produce have varying physical properties and compositions, leading to different particle sizes. The pyrolysis process, including temperature, also affects particle size; higher temperatures usually result in finer particles due to the thorough decomposition of biomass. Additionally, physical modifications, such as crushing and sieving of the obtained biochar or the addition of magnetic properties or coatings, can further influence the particle size. For example, the obtained corncob biochar was crushed and sieved to <0.15 mm.

### 3.2. Activated Carbon

Activated carbon is biochar that has undergone chemical or physical activation using heat and other chemical agents [51,59]. Within the last 10 years, 28% of published papers have targeted the use of activated carbon as an adsorbent to remove MPs from wastewater, and this research actively started around 2019, as seen in Figure 5. Activated carbon traps MPs through physical adsorption, and the large surface areas and porous structures provide several active sites that can trap MPs. The adsorption is driven by van der Waals forces and hydrophobic interactions between the MPs and the carbon surface [60,61].

#### 3.2.1. Carbon Content of Activated Carbon

Out of 925 research papers reviewed, none mentioned the presence of fixed carbon nor the elemental carbon present in activated carbon. Even though the removal efficiency of activated carbon is generally high, above 90%, the carbon content in the adsorbent was not mentioned [47,48,62,63].

#### 3.2.2. Surface Area of Activated Carbon

Four papers also revealed the surface areas of the adsorbent. Activated carbon is generally known for its extremely high surface area, often ranging from 600 m^2^/g to over 1000 m^2^/g, as seen in Table 2. This high surface area provides a great number of adsorption sites. Generally, it can be seen that a high surface area is directly correlated with a high adsorption efficiency.

For example, granular activated carbon also shows high efficiency, 95.5%, with a relatively high surface area of 682.24 m^2^/g [47]. Activated carbon had a moderate surface area of about 488 m^2^/g but still showed a very high efficiency of between 89 and 100% [63]. CuNi carbon material had the lowest surface area of 110.48 m^2^/g but showed a very high efficiency of 99.18% [48]. The efficiency of activated carbon is generally positively correlated with its surface area. However, surface area alone is not the sole determinant of adsorption efficiency. The type of activation, pore size distribution, and chemical properties of the activated carbon also play crucial roles in determining its efficiency.

#### 3.2.3. Pore Size of Activated Carbon

Five papers mentioned the pore size of the adsorbent. Activated carbon has a well-developed pore structure with a mix of micropores and mesopores, as seen in Table 2. Pore sizes have been reported in the range of 1.88 nm to 15 µm, which is effective in the trapping of MPs. Activated carbon with a combination of micropores and mesopores generally showed high adsorption efficiencies. For example, granular activated carbon with micropores and mesopores of 1.88 nm achieved 95.5% efficiency [47]. It was also observed that larger pore sizes with mesopores showed a high adsorption efficiency, with a pore size of 5.673 nm showing removal efficiencies between 89 and 100% [63]. It was also observed that granular activated carbon with a wide range of mesopores and macropores achieved a high adsorption efficiency of 98% [61]. Different activation methods (e.g., phosphoric acid and zinc chloride) resulted in different efficiencies, indicating that the chemical and physical properties imparted by the activation process are crucial [64]. The efficiency of activated carbon for adsorption is significantly influenced by the type and size of the pores.

#### 3.2.4. Particle Size of Activated Carbon

Two papers also mentioned the particle size of the carbon adsorbent. It was observed that a higher removal efficiency was obtained with smaller particle sizes, as seen in Table 2. For example, porous activated carbon with particle sizes between 0.5 mm and 1 mm showed a very high efficiency of 217.39 mg/g [64]. Granular activated carbon with particle sizes between 1.16 mm and 2.5 mm also showed a high efficiency of 95.5% [47].

The activation method and surface properties of the activated carbon also play significant roles in determining the efficiency, indicating that particle size is important but not the sole factor in adsorption performance [64].

### 3.3. Carbon Nanotubes

The development of carbon-based adsorbents such as carbon nanotubes has become an emerging topic for research in recent years, as seen in Figure 5. Within the last 10 years, 7% of published papers targeted the use of carbon nanotubes as an adsorbent to remove MPs from wastewater, and this research actively started around 2019. Carbon nanotubes adsorb MPs through hydrophobic interactions and van der Waals forces [65,66].

#### 3.3.1. Carbon Content of Carbon Nanotubes

Out of 232 research papers reviewed, only one paper took into account the carbon content of the adsorbent. A high carbon content typically correlates with good structural properties and high adsorption capacities. CNTs have very high carbon content, usually above 90%. For example, a carbon content of 93.37% was observed in magnetic carbon nanotubes [49].

#### 3.3.2. Surface Area of Carbon Nanotubes

One paper also considered the surface area of the adsorbent. The surface area of CNTs is also very high. For example, a surface area of 197.186 m^2^/g is substantial and plays a crucial role in the high adsorption efficiency (100%) observed. A greater surface area means that more adsorption sites are available for interaction with contaminants [49].

#### 3.3.3. Pore Size of Carbon Nanotubes

CNTs are made up of well-defined mesopores, typically about 2 nm to 4 nm, which are effective in microplastic adsorption. The presence of mesopores (2.857 nm) in magnetic carbon nanotubes contributes significantly to their high adsorption efficiency [49]. Mesopores provide optimal conditions for adsorbing a variety of molecules, enhancing the effectiveness of the carbon nanotubes.

#### 3.3.4. Particle Size of Carbon Nanotubes

Specific particle sizes for CNTs are often not reported. A small particle size (diameter 1–2 nm) enhances the adsorption efficiency by increasing the available surface area and improving the dispersion of the adsorbent [49].

### 3.4. Graphene

The development of carbon-based adsorbents such as carbon nanotubes has become a hot topic in recent years, as seen in Figure 5. Within the last 10 years, 8% of published papers targeted the use of graphene-based adsorbents to remove MPs from wastewater, and this research actively started around 2020. Graphene is suitable for microplastic removal because it has a large surface area, which offers numerous active sites for adsorption. Strong π–π interactions between graphene and microplastics ensure that they bind tightly. The oxygen-containing functional groups in graphene add extra interaction sites, enhancing adsorption [67]. Additionally, graphene’s hydrophobic nature complements these π–π interactions, making the adsorption process even more effective [68].

#### 3.4.1. Carbon Content of Graphene

Out of 268 research papers reviewed, only one paper took into account the carbon content of the adsorbent. Graphene-based adsorbents also have a high carbon content, as seen in Table 3. Although the specific carbon content data for the carbon-carbon nanofiber were not mentioned, it was observed that graphite carbon nitride, with a moderate carbon content of 40.08–46.23%, showed a very high efficiency of 99.32% [50].

#### 3.4.2. Surface Area of Graphene

Only two papers took into account the surface area of the adsorbent. Graphene-based adsorbents are characterized by a very high surface area, as seen in Table 3. High surface area proves beneficial for adsorption.

For instance, graphite carbon nitride with a 1090 m^2^/g surface area showed 99.32% efficiency [50] compared to the carbon-carbon nanofiber (732.2 m^2^/g), with an efficiency range of 93.2–98.5% [69].

#### 3.4.3. Pore Size of Graphene

Two papers reported the pore size of the adsorbent. Graphene-based adsorbents present micropores and mesopores with a typical pore size of about 2–4 nm, as seen in Table 3. Smaller mesopores (1.88–2.34 nm) in graphite carbon nitride are associated with slightly higher efficiency [69] compared to the slightly larger pore range (2–4 nm) in the carbon-carbon nanofiber [50]. Mesopores are effective for adsorbing small molecules, contributing to high efficiency.

#### 3.4.4. Particle Size of Graphene

One paper mentioned the particle size of the adsorbent. In Table 3, smaller particle sizes (40–60 nm) in graphite carbon nitride likely enhance the adsorption efficiency by increasing the surface area and providing better interactions with MPs [50].

## 4. Discussion

### 4.1. Adsorption Mechanisms

Physical and chemical interactions are two of the dominant mechanisms controlling the adsorption of MPs onto carbon-based adsorbents, including biochar, activated carbon, graphene, and CNTs [48]. Specific surface area, pore size distribution, and particle size have been critical in defining the effectiveness of these adsorbents.

Specific Surface Area: A higher specific surface area provides more active sites to the adsorbent which microplastics can bind to, thereby improving their adsorption capacity. For example, graphite carbon nitride with a surface area of 1090 m^2^/g is 99.32% efficient in adsorbing microplastics [50]. Activated carbon with a surface area of 682.24 m^2^/g is 95.5% efficient in adsorbing microplastics [47]. This is because the high surface area facilitates more complete contact between the adsorbent and microplastic particles in wastewater.

Pore Size Distribution: The pore size of an adsorbent usually affects the characteristics of the trapping of MPs in a specified size range. Larger microplastic particles can be captured by macropores, while smaller fractions, like microfibers or nano-sized MPs, can only be better removed with mesopores and micropores. Pore sizes of between 3 nm and 30 nm make biochars efficient adsorbents in trapping bigger microplastics [43,46,55]. While, for instance, biochar is characterized by a more widely distributed pore size that allows for the trapping of microplastics of diverse dimensions, microporous structures are more appropriate for small MPs. Activated carbon, graphene, and carbon nanotubes have small pore sizes ranging between 1.88 nm and 2.857 nm, making them more suitable to capture smaller MPs [49,50,64].

Particle Size: The reduction in the size of particles provides higher surface area-to-volume ratios and, hence, an enhancement in the rate of adsorption. Activated carbon and biochar particles are larger, ranging between 1 mm and 2 mm, and hence are easier to filter [47,55]. Graphene and CNTs had smaller particle sizes ranging between 1 nm and 60 nm and were more adsorptive, with efficiencies greater than 99.32% [49,50].

Apart from physical properties, chemical interactions also play an important role in the adsorption of MPs. For non-polar MPs, the major interaction mechanisms with carbon-based materials are provided by van der Waals forces and hydrophobic interactions. Since the adsorbate molecules contain weak van der Waals forces, hydrophobic interactions take place as the molecules gather, thus holding off water molecules. This adsorption is possible because of the absence of strong covalent, ionic, and hydrogen bonds [70]. Hydrophobic interactions drive the adsorption process, effectively improving microplastic removal [63].

Moreover, the presence of oxygen-containing functional groups like graphene oxide on the material surface promotes the adsorption of polar microplastics through hydrogen bonding and electrostatic interactions [71]. It was seen that the oxygen-holding functional groups of biochar resulted in the effective adsorption of microplastics [58].

Recent research has also indicated that there could be π–π interactions between the aromatic rings in microplastics and carbon structures in graphene or activated carbon. For example, the adsorption mechanism of graphene was mainly attributed to the strong π–π interaction between the carbon ring of the graphene oxide and the benzene ring of the microplastic [68]. This π–π interaction, along with hydrophobic effects, enhances the adsorption performance of advanced carbon materials substantially [49].

### 4.2. Advantages and Disadvantages of Different Types of Carbon Adsorbents

Carbon-based adsorbents, including biochar, activated carbon, CNTs, and graphene, have been widely recognized for their effectiveness in removing MPs from wastewater. Each adsorbent type has distinct advantages and disadvantages, which are summarized in Table 4.

### 4.3. Comparative Analysis of Adsorbents

A comparative analysis based on the physical and chemical properties of different types of adsorbents is essential to determine their effectiveness in microplastic removal.

Biochar is an economical and eco-friendly adsorbent, and it turns out to be viable as it is derived from waste biomass [76]. The surface area of biochar ranges from 175 m^2^/g to 700 m^2^/g, making it moderately effective in microplastic removal. Biochar has a different pore size distribution, which might capture MPs of various sizes [55]. However, due to the low surface area and lower porosity, its adsorption capacities typically tend to be relatively lower compared with other adsorbents [46]. Biochar efficiency could be very different depending on the biomass source, modification, and production conditions such as pyrolysis temperature. For example, corn straw biochar had a range of efficiencies (68.7–81.3%) dependent on pyrolysis temperature [42]. Modifications can effectively improve the efficiency of biochar [77]. The adsorption efficiency of sawdust biochar modified with magnetization varies within the range of 94.81–99.46%, compared to unmodified biochar with an efficiency of 25.89% [43]. The principal advantages of biochar relate to its low cost and sustainable use on a large scale.

Activated carbon is particularly known for its extremely high surface area, hence providing numerous active sites for adsorption. This high surface area results in a high adsorption capacity, making activated carbon a well-established material in water treatment processes. For instance, the removal efficiency of granular activated carbon is as high as 95.5%, while its surface area is up to 682.24 m^2^/g [47]. Activated carbon has a micro-porous structure that could effectively adsorb both the relatively smaller microplastic particles and even nanoparticles. However, its performance is highly dependent upon pore size distribution. Because of this, some micropores, which may be well developed within the structure of activated carbon, can effectively trap the smaller MPs, like synthetic microfibers, with larger particles posing difficulties in trapping. Compared with the biochar, activated carbon is much more expensive due to its energy-intensive production process. Furthermore, a regeneration process of activated carbon will be challenging and costly, which makes reusing it a very limited option.

With their high surface area and well-defined pore structure, CNTs can be one of the best adsorbents. For example, magnetic CNTs possess a surface area of 197.186 m^2^/g and 100% adsorption efficiency [49]. They demonstrate very good mechanical properties, stability and regeneration; hence, they seem very effective in the removal of both small and large MPs [62]. The tubular structure allows the possibility of running several mechanisms of adsorption, including van der Waal forces, hydrophobic interactions, and electrostatic attractions. However, CNTs are still problematic due to their high production costs and complex processes, which make CNTs much less accessible for broad applications [78].

Graphene is an excellent adsorbent due to its distinctive structure and π–π interactions, which form strong chemical bonds with microplastics [79]. These provide a high surface area and excellent adsorption capacity. For instance, graphite carbon nitride has a surface area of 1090 m^2^/g and presents an efficiency of 99.32% [50]. Graphene is effective in many types of pollutants. However, the large-scale production and application of graphene is still emerging, thus limiting MP removal applications [80].

## 5. Challenges, Future Perspectives, and Conclusions

Over the past few years, extensive research has been carried out on the use of carbon-based adsorbents to remove microplastics from wastewater. Although some achievements have been made, there is still room for improvement. One of the big challenges was that clear comparisons were not available to show how the various adsorbents compared to each other in efficiency and performance. This lack of comparison data made it difficult to determine which was the best adsorbent for given applications. Various studies did not compare the results using standardized methods; hence, there is a need to develop standardized protocols so that the data obtained will be reliable and comparable. The variable efficiency of biochar was the critical issue, and it remained dependent on the biomass source and conditions of pyrolysis. For example, the efficiency of corn straw biochar ranged from 68.7% to 81.3%, depending on the pyrolysis temperature; thus, its standardization was difficult. Also, the regeneration of activated carbon was usually cumbersome. Ensuring that adsorbents can be re-used without significant efficiency loss is crucial for economic viability.

Future efforts should be directed at economically viable and scalable production methods of advanced adsorbents like graphene and CNTs, so that such high-efficiency materials can be put to task easily for real-scale applications. The optimization of the production of biochar requires further research to ensure that a high adsorption efficiency will always be offered. The standardization of pyrolysis conditions and investigations of a wide range of feedstocks would probably make the production of biochar-based adsorbents more feasible. The use of modification methods, such as magnetically active or polymer-based coatings, could substantially improve the performance of biochar. For example, several researchers have modified sawdust biochar to enable it to possess magnetic properties, and they achieved very high efficiencies ranging between 94.81% and 99.46%, hence showing good prospects for such modifications. Also, future work could develop methods to implement advanced adsorbents within appropriate water treatment systems. A combination of physical, chemical, and biological techniques with advanced adsorbents could give better overall MP removal efficiency. Such is the case where a combination of biochar with membrane filtration or advanced oxidation processes results in enhanced MP removal.

The main mechanisms by which carbon adsorbents trap microplastics include hydrophobic interactions, van der Waals forces, π–π interactions, and electrostatic interactions. Particle size, pore size, surface area, and carbon content affect the ability of adsorbents to trap MPs. With an in-depth understanding of the relationship between the surface characteristics of adsorbents, one will be able to design optimized adsorbents for MP wastewater removal with high efficiency and thus contribute much to environmental protection and public health.

## Figures and Tables

**Table 1:** Surface characteristics of biochar.

Adsorbent Used	Adsorption Efficiency (%)/Concentration (mg/g)	Carbon Percentage	Surface Area (m^2^ g^−1^)	Average Pore Size	Particle Size Range	References
Corn straw biochar @ 300 °C	95%	70.0%	808.3	Not mentioned	0.6–0.7 mm	[42]
Corn straw biochar @ 400 °C	95%	81.3%	609.0		0.6–0.7 mm	
Corn straw biochar @ 500 °C	95%	68.7%	177.5		0.6–0.7 mm	
Hardwood biochar	95%	84.7%	292.8		0.6–0.7 mm	
Sawdust biochar @ 550 °C (no modification)	25.89%	Not mentioned	Not mentioned	1.86 nm	Not mentioned	[43]
Sawdust biochar @ 550 °C (magnetic biochar)	94.81%	69.29%	363.80	3.81 nm		
Sawdust biochar @ 550 °C (Mg modified magnetic biochar)	98.75%	65.03%	265.74	3.83 nm		
Sawdust biochar @ 550 °C (Zn modified magnetic biochar)	99.46%	66.48%	329.87	3.83 nm		
Rape straw biochar @ 600 °C (CTAB modified magnetic biochar)	91.2–95%	66.99%	683.6	Not mentioned	Not mentioned	[44]
Activated biochar @ 850 °C	Not mentioned	66.1%	897.7	10.83 nm	30.56 nm	[45]
Magnetic activated biochar @ 650 °C	96–100 mg/g	50.6%	926.69	9.82 nm	20.68 nm	
Polymer-coated magnetic activated biochar @ 650 °C	99–100 mg/g	42.8%	Not mentioned	Not mentioned	Not mentioned	
Corncob biochar @ 500 °C	12 mg/g	76.80%	17.8	7.31 nm	<0.15 mm	[46]
Corncob biochar @ 700 °C	14 mg/g	79.90%	34.5	9.12 nm	<0.15 mm	
Corncob biochar @ 900 °C	15 mg/g	81.20%	36.6	9.79 nm	<0.15 mm	
Corncob biochar @ 500 °C (oxidized)	14 mg/g	74.70%	36.9	7.07 nm	<100 nm	
Corncob biochar @ 700 °C (oxidized)	16 mg/g	72.90%	48.2	8.82 nm	<100 nm	
Corncob biochar @ 900 °C (oxidized)	18 mg/g	65.30%	60.8	9.09 nm	<100 nm	
Walnut shell biochar @ 700 °C	0.27–0.79 mg/g	86.44%	512.18	3.23 nm	150 μm	[54]
Iron modified Walnut shell biochar @ 700 °C	0.77–6.75 mg/g	59.27%	892.64	3.17 nm	150 μm	
Biochar @ 450 °C	68.2 mg/g	45%	39.4	30.13 nm	1–2 mm	[55]
Biochar @ 500 °C	86.78 mg/g	48%	49.2	17.17 nm	1–2 mm	
Biochar @ 550 °C	89.06 mg	50%	49.2	13.04 nm	1–2 mm	
Biochar @ 550 °C	290.20 mg/g	34.05%	25.8	Not mentioned	Not mentioned	[56]
Biochar @ 850 °C	206.46–225.11 mg/g	46.42%	34.5			
Jujube waste biochar @ 300 °C	98%	Not mentioned	Not mentioned	Not mentioned	0.5 mm	[57]
Jujube waste biochar @ 700 °C	98%	Not mentioned	Not mentioned		0.5 mm	
Banana peel biochar	92.16%	Not mentioned	12.277	Not mentioned	0.15–0.5 mm	[58]

**Table 2:** Surface characteristics of activated carbon.

Adsorbent Used	Adsorption Efficiency (%)/Concentration (mg/g)	Surface Area (m^2^ g^−1^)	Average Pore Size	Particle Size Range	References
Granular activated carbon	95.5%	682.24	1.88 nm	1.16–2.5 mm	[47]
CuNi carbon material	99.18%	110.48	8.25 nm	Not mentioned	[48]
Granular activated carbon	98%	Not mentioned	15 μm–100 nm	Not mentioned	[61]
Granule-activated carbon	92.8%	Not mentioned	Not mentioned	Not mentioned	[62]
Activated carbon	89–100%	488	5.673 nm	Not mentioned	[63]
Porous activated carbon using phosphoric acid	217.39 mg/g	Not mentioned	2.65 nm	0.5–1 mm	[64]
Porous activated carbon using zinc chloride	73.53 mg/g	Not mentioned	3.01 nm		

**Table 3:** Surface characteristics of graphene.

Adsorbent Used	Adsorption Efficiency (%)/Concentration (mg/g)	Carbon Content	Surface Area (m^2^/g)	Average Pore Size	Particle Size Range	References
Graphite carbon nitride	99.32%	40.08–46.23%	1090	1.88–2.34 nm	40–60 nm	[50]
Carbon-based graphite carbon-carbon nanofiber	93.2–98.5%	Not mentioned	732.2	2–4 nm	Not mentioned	[69]

**Table 4:** Advantages and disadvantages of carbon adsorbents.

Adsorbent	Advantages	Disadvantages	References
**Biochar**	- Cost-effective and widely available; - High surface area and porous; - Can be produced from various biomass sources; - Environmentally friendly.	- Lower adsorption capacity compared to more advanced materials like activated carbon; - Efficiency varies based on feedstock and production conditions; - Often requires modification to enhance properties.	[72]
**Activated Carbon**	- Known for its high adsorption capacity; - Large surface area; - Effective across a wide range of pollutants.	- Higher cost compared to biochar; - High energy consumption during production; - Regeneration can be challenging.	[73]
**CNTs**	- Extremely high surface area and adsorption efficiency; - Excellent mechanical properties.	- High production costs; - Complex manufacturing in large-scale applications.	[74]
**Graphene**	- Very high surface area; - Strong adsorption potential due to unique π–π chemical properties.	- Expensive to produce; - Challenges in large-scale production and application.	[75]

## Data Availability

No new data were created or analyzed in this study.
